# Effectiveness of HBV Vaccination in Infants and Prediction of HBV Prevalence Trend under New Vaccination Plan: Findings of a Large-Scale Investigation

**DOI:** 10.1371/journal.pone.0047808

**Published:** 2012-10-19

**Authors:** Shi-gui Yang, Bing Wang, Ping Chen, Cheng-bo Yu, Min Deng, Jun Yao, Chun-xia Zhu, Jing-jing Ren, Wei Wu, Bin Ju, Jian-feng Shen, Yu Chen, Ming D. Li, Bing Ruan, Lanjuan Li

**Affiliations:** 1 The State Key Laboratory for Diagnosis and Treatment of Infectious Diseases, The First Affiliated Hospital, School of Medicine, Zhejiang University, the Key Laboratory of Infectious Diseases, Zhejiang Province, Hangzhou, China; 2 Zhejiang Provincial Center for Disease Control and Prevention, Hangzhou, China; 3 Zhejiang Province Health Bureau Center of Information, Hangzhou, China; Centers for Disease Control and Prevention, United States of America

## Abstract

**Background:**

Hepatitis B virus (HBV) infection remains a severe public health problem. Investigating its prevalence and trends is essential to prevention.

**Methods:**

To evaluate the effectiveness of HBV vaccination under the 1992 Intervention Program for infants and predicted HBV prevalence trends under the 2011 Program for all ages. We conducted a community-based investigation of 761,544 residents of 12 counties in Zhejiang Province selected according to their location, population density, and economic development. The HBV prevalence trends were predicted by a time-shifting approach. HBV surface antigen (HBsAg) and alanine amino transferase (ALT) were determined.

**Results:**

Of the 761,544 persons screened for HBsAg, 54,132 were positive (adjusted carrier rate 6.13%); 9,455 had both elevated ALT and a positive HBsAg test (standardized rate 1.18%). The standardized HBsAg carrier rate for persons aged ≤20 years was 1.51%. Key factors influencing HBV infection were sex, age, family history, drinking, smoking, employment as a migrant worker, and occupation. With the vaccination program implemented in 2011, we predict that by 2020, the HBsAg carrier rate will be 5.27% and that for individuals aged ≤34 years will reach the 2% upper limit of low prevalence according to the WHO criteria, with a standardized rate of 1.86%.

**Conclusions:**

The national HBV vaccination program for infants implemented in 1992 has greatly reduced the prevalence of HBV infection. The 2011 program is likely to reduce HBV infection in Zhejiang Province to a low moderate prevalence, and perinatal transmission is expected to be controlled by 2020.

## Introduction

Hepatitis B virus (HBV) infection is a serious problem worldwide[1], [2]. Approximately 2 billion infected people are alive, of whom 350 million have chronic infection, 75% reside in the Asia Pacific region, and about 1 million per year die[3], [4], [5], [6].In China, the HBV surface antigen (HBsAg) carrier rate was 8.75% in 1979[7], 9.75% in 1992[8], and 7.18% in 2006[9].

Because it is the most effective prevention strategy[10], [11], an HBV vaccination program for infants was implemented in China in1992 (1992 Program) and caused a significant decline in HBV prevalence[9]. However, its benefit: cost ratio and the current HBV prevalence are unknown. Moreover, it has been more than 5 years since the last national seroepidemiologic survey of hepatitis B in China. During this period, significant changes in the economy, population density, and health conditions have occurred, especially in Zhejiang Province on the southeast coast, which is highly developed economically and has a dense population with many migrant workers and a higher than average HBsAg carrier rate (7.7% vs. 7.18% for the country)[9].

Thus, we carried out a community-based epidemiologic investigation of hepatitis B among 761,544 people from Zhejiang Province with the following objectives: 1) to determine the current HBV prevalence rate and predisposing factors for infection; 2) to evaluate the effectiveness of the national 1992 HBV vaccination program; and 3) to predict the trend in HBV prevalence with an all-ages vaccination program implemented in 2011. Finally, we conducted a benefit: cost analysis on the 2011 HBV vaccination program in comparison with the one implemented in 1992.

## Methods

### Study Population and Data Collection

The health exam plan was first implemented by Zhejiang Province in 2005 and has been provided to all residents of the province every two years since then for free of charge. The plan includes physical examination (e.g., interrogation, auscultation, measuring blood pressure, etc) and routine laboratory testing (such as levels of blood glucose and blood lipids etc). With the support of the Mega-Project for National Science and Technology Development for the “11th Five-Year Plan of China” and the Department of Health of Zhejiang Province, HBsAg test and alanine aminotransferase [ALT] assays were added to the plan in 12 counties of the province, in 2010. Twelve counties, home to about 10% of the provincial population, were chosen by considering geographic location, population density, and economic development throughout the province.

After receiving appropriate training by leading researchers for this study, the physicians of each participating hospital began to conduct medical examinations, interviews, and laboratory tests on subjects who volunteered for the free medical and health examinations. Together, approximately 3,700 physicians from 168 hospitals in the twelve counties were invited by the Department of Health of Zhejiang Province to engage in the recruitment of participants, interviewing, and/or medical examinations. All local residents and migrant workers who had lived in the area for >3 months (i.e., moved to the area before September 1, 2009) were qualified for inclusion; the final average participation rate was about 60% among all the participating sites. The information collected consisted of demographics (sex, age, occupation, nationality, and marital status), residency status, habits (smoking, drinking, diet, and extent of physical exercise), medical history (e.g., blood transfusion, hepatitis B, allergies), and laboratory tests (HBsAg and alanine aminotransferase [ALT] assays). The consent statement was in written form and the informed written consent from the next of kin on the behalf of the minors/children participants. The study was approved by the Ethics Committee of The First Affiliated Hospital at the School of Medicine of Zhejiang University.

Each participating physician or his/her designee entered all the information in a password-protected, province-wide Electronic Health Records (EHR) system, which was developed by the Department of Health of Zhejiang Province to store and manage medical records and laboratory-testing results for all residents of the province who participated the province-wide health examination plan. By examining the physical and interview data for 1,172,218 residents of the 12 counties collected between January 1 and December 31, 2010, we identified 761,544 participants with both ALT and HBsAg testing results.

To confirm the accuracy and reliability of the EHR data, we conducted an independent epidemiologic sampling of the prevalence of HBsAg carriers and hepatitis B according to the three-stage stratified design. For each selected county, we randomly selected three towns to survey a minimum of 2000 subjects per town based on age groups with different carrier rates. For this stage, we recruited a total of 72,254 subjects into the validation study. All subjects were directed to fill out questionnaires and provide a 5-mL blood sample for HBsAg and ALT tests in a central facility. The kits used for HBsAg and ALT tests were purchased from the government-certified manufacturers with sensibility of 99.73% and specificity of 99.9% for HBsAg assessment, and International Federation of Clinical Chemistry (IFCC) for Alanine Aminotransferase (ALT).

### Vaccination Programs

#### Intervention for HBV prevention in infants (“1992 Program”)

In order to reduce HBV infection rate in China, a national HBV vaccination program for all infants was implemented by the Chinese government in 1992. Under this program, the first vaccine dose was administered within 24 hours of birth and subsequent doses at 1 and 6 months[9]. A recombinant HBV vaccine at a concentration of 5 µg/dose was given. Supplementary immunization activities were conducted for children born after January 1, 1992 who did not get vaccination or finish the program[12]. The 1992 vaccination program did not include HBIG (hepatitis B immunoglobulin) use. From 1992 to 2010, average vaccination coverage rate for infants in these 12 counties were from 91.70% to 99.90%.

#### Intervention for HBV prevention in both infants and adults (“2011 Program”)

To further reduce the overall HBV infection rate, a new program not only for infants but also for adults born before 1992 (which were not covered under the 1992 Program) was implemented in the selected 12 counties in 2011with a higher vaccine dose (10 µg) as recommended by the National Expert panel for the Mega-Project for National Science and Technology Development in China. For infants, the vaccine was given on the same schedule as in the earlier program. For adults negative for HBV antigens and antibodies, an identical immunization program was adopted.

### Statistical Analysis

All data were stored in an Oracle database and analyzed using SPSS for Windows (release 13.0). For categorical variables, we used the chi-square test to determine group differences. The HBsAg carrier rate was standardized by age and sex according to the 2010 Zhejiang population structure[13]. Stratified analysis was used for determination of risk factors for HBV infection.

According to the 2010 population structure of Zhejiang Province and under the assumption of the same life expectancy for the population in 2020, we predicted the prevalence trend of HBV infection for 2020 with a time-shifting approach. In our prediction model, *Y_i_* stands for age *I* and *in*-*Y_i_* stands for the population under age *i*. The HBsAg carrier rate for in-Yi (%)  =  number of HBsAg carriers *in*-*Y_i_* /number of persons tested for HBsAg *in*-*Y_i_*. Cumulative population percentage *in*-*Y_i_*  =  summary of the percentage of each year of age in *Y_i_*. The standardized average carrier rate in *Y_i_* (%) was calculated by the average carrier rate *in*-*Y_i_* standardized by the 2010 Zhejiang population structure. Under the 2011 Program, the average emerging infection rate (%) of population *in*-*Y_i_*  =  HBsAg-negative rate × HBsAb-negative rate in the HBsAg-negative population × failure rate of immune protection × emerging infection rate of the 1992 Program. The average carrier rate (%) of population *in*-*Y_i_* by 2020 was calculated according to the average carrier rate of the population under age *Y_i_*-10 in 2010 plus the average emerging infection rate of population *in*-*Y_i_*. All data on the HBsAg carrier rate in 1992 and the coverage rate of HBV vaccination of infants in Zhejiang Province were adopted from the literature[14], [15], [16]. In the reported 1992's study[15], a total of 12 districts with 400 persons included per district in the province of Zhejiang were selected by using stratified multistage random cluster sampling.

The cost and benefit analyses, based on the annual background mortality rate and HBV-specific cause costs, were conducted as described [17]and can be summarized briefly as follows: Cost: effectiveness ratio  =  total cost for testing and vaccination/reduced number of patients with chronic hepatitis B (CHB), cirrhosis, or hepatocellular carcinoma (HCC); cost: utility ratio  =  total cost for testing and vaccination/avoided disability-adjusted life years (DALY); benefit: cost ratio  =  total benefits in saving treatment for CHB, cirrhosis, and HCC / total cost for testing and vaccination. For a detailed description of these analyses, please see Supplementary Table 1.

**Table 1:** HBsAg carrier rate and prevalence of suspected hepatitis Bin male and female samples.

Classification	Sex (Sample size)	Positives (N)	Crude Rate (%)	Standardized Rate by Age (%)	Sp (%)	95% CI	P value
HBsAg	Male (312,763)	25,509	8.16	7.33	0.05	7.24, 7.42	<0.01
	Female (439,669)	27,982	6.36	5.34	0.03	5.27, 5.41	
Suspected Hepatitis B	Male (299,040)	5,660	1.89	1.82	0.02	1.78, 1.87	<0.01
	Female (419,630)	3,682	0.88	0.75	0.01	0.73, 0.78	

## Results

### HBsAg Carrier Rate and Suspected Hepatitis B Prevalence

Of the 761,544 subjects, 312,763 were male; 32,557 were ≤20 years, 509,415 were 20–59 years old, and 219,572 were ≥60 years. Of the 761,544 persons screened for HBsAg, 54,132 (7.11%) were positive, with a standardized (adjusted by sex and age) HBsAg carrier rate of 6.13% (95% confidence interval [CI] 6.08, 6.19). Of the 727,746 subjects screened for both HBsAg and ALT, 9,455 (1.3%) had both indicators of hepatitis, with a standardized rate of 1.18% (95% CI 1.16, 1.21).

Among male subjects, the crude HBsAg carrier rate and prevalence of suspected hepatitis B were 8.16% and 1.89%, respectively, and the standardized rate was 7.33% (95% CI 7.24, 7.42) and 1.82% (95% CI 1.78, 1.87), respectively (Table 1). Among female subjects, the crude HBsAg carrier rate and the prevalence of suspected hepatitis B were 6.36% and 0.88%, respectively, the standardized rates being 5.34% (95% CI 5.27, 5.41) and 0.75% (95% CI 0.73, 0.78), respectively.

**Figure 1:**
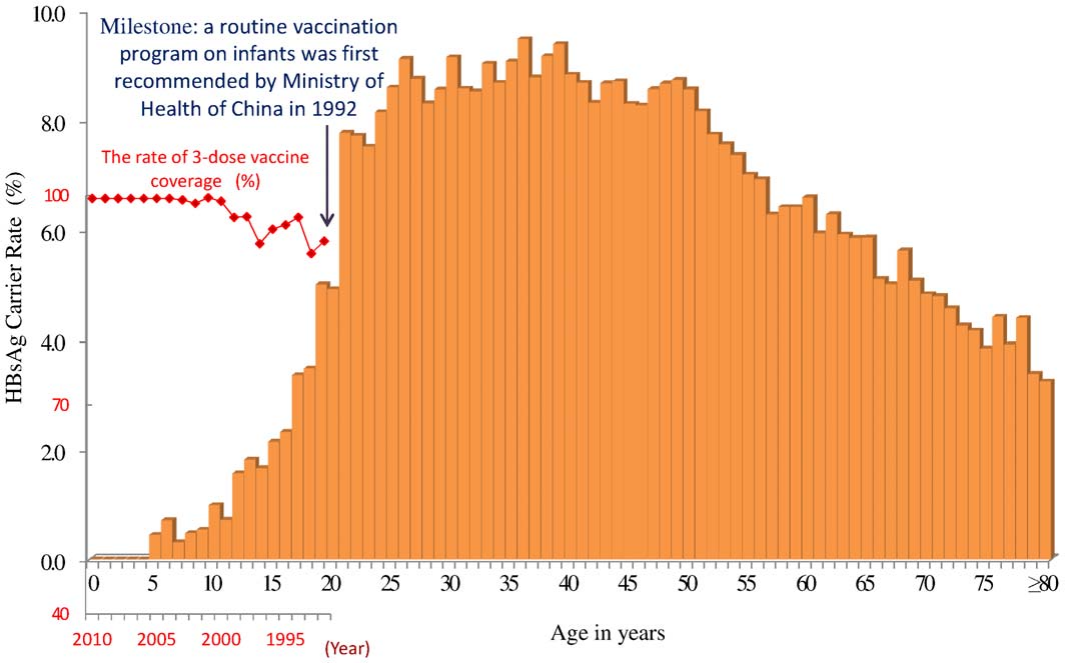
Distribution of HBsAg carrier rate by age. All participants were included in the community-based EHR, which contained test results for 761,544 participants. As shown here, there was a dramatic reduction in the HBsAg rate among individuals born after 1992, when a national neonatal HBV vaccination program was implemented. Prior to 1992, however, the HBsAg carrier rate was as high as 8%.

As expected, we found significant differences by age in the HBsAg carrier rate and prevalence of suspected hepatitis B (Fig. 1). Among the subjects <20 years, the HBsAg carrier rate was significantly lower than in older subjects: crude rate 2.74% and standardized rate by sex 1.51%; for middle-aged subjects (20–59 years old) average crude HBsAg carrier rate 8.15% and standardized rate by sex 8.22%; for subjects ≥60 years HBsAg carrier rate 5.07% and standardized rate by sex 4.96%.

We believe the significantly reduced HBsAg carrier rate among the young is attributable to the vaccination of infants beginning in 1992. Similarly, the prevalence of suspected hepatitis B in subjects <20 years was low (0 to 0.6%) and was highest among persons aged 30–39 years (1.88%) (Fig. S1).

### Factors Influencing HBsAg Carrier Rate and Prevalence of Hepatitis B in Persons 20–60 Years Old

The factors identified as influencing the HBsAg carrier rate included working as a long-distance truck driver (relative risk [RR]1.53; 95% CI 1.27, 1.86) or in construction (RR1.58; 95% CI 1.34, 1.86), being a migrant worker (RR1.15; 95% CI 1.01, 1.31), having a family history of hepatitis B (RR1.47; 95% CI 1.39, 1.55), smoking (RR1.36; 95% CI 1.33, 1.40), drinking (RR 1.30; 95% CI 1.27, 1.34), or living in a coastal region (RR1.87; 95% CI 1.84, 1.91) (Table 2). Male truck drivers and migrant workers howed a significantly higher HBsAg carrier rate than females in the same categories.

**Table 2:** Factors influencing HBsAg carrier rate for ages 20 to 60 years.

Influencing Factors	Standardized Rate % (N)		RR	95% CI	ARP* (%)	P Value
	Exposure	Non-exposure				
Long-distance truck drivers	12.69 (731)	8.28 (636,957)	1.53	1.27, 1.86	10.39	<0.01
Construction workers	13.07 (933)	8.28 (636,755)	1.58	1.34, 1.86	36.70	<0.01
Migrant workers	7.35 (2,989)	6.39 (586,288)	1.15	1.01, 1.31	13.04	<0.05
With family history	9.42 (13,746)	6.41 (433,926)	1.47	1.39, 1.55	31.97	<0.01
Smoking	11.29 (70,876)	8.28 (292,116)	1.36	1.33, 1.40	26.47	<0.01
Drinking	10.97 (62,156)	8.41 (300,177)	1.30	1.27, 1.34	23.08	<0.01
Residents in coastal areas	9.07 (249,941)	4.84 (488,472)	1.87	1.84, 1.91	46.52	<0.01

* Notes:Attributable risk proportion =  proportion of increased risk attributable to the factor.

Next, we identified the factors contributing to the high prevalence of hepatitis B, which included being a construction worker (RR1.68; 95% CI 1.15, 2.45), having a family history of hepatitis B (RR1.74; 95% CI 1.56, 1.95), smoking (RR1.83; 95% CI 1.74, 1.93), drinking (RR1.89; 95% CI 1.79, 1.99), or living in coastal regions (RR2.38; 95% CI 2.28, 2.48). A multivariate stratified analysis revealed that males and individuals aged ≥20 years (i.e., those not covered under the 1992 Program) had a significantly higher prevalence of hepatitis B if they were construction workers or had a smoking or a family history of hepatitis B (Table S2).

There was a high prevalence of hepatitis B in persons living in coastal regions, especially if they were fishermen (Fig. S2A). This was especially true among young and middle-aged (20–49 years) men, where the HBsAg carrier rate was as high as 14.95% (range 14.34%–15.84%), and the prevalence of suspected hepatitis B was 4.48% (Fig. S2B). Moreover, the HBsAg carrier rate (14.62%) and the prevalence of hepatitis B (4.24%) were significantly higher among fishermen than in non-fishermen (7.07% and 1.29%, respectively). This was especially true for male fishermen aged 20–49 years, where the HBsAg carrier rate and prevalence of hepatitis B were 17.53% and 6.05%, respectively (Fig. S2C, D).

### Developing Trend of Hepatitis B Prevalence under the HBV Vaccination Programs

**Figure 2:**
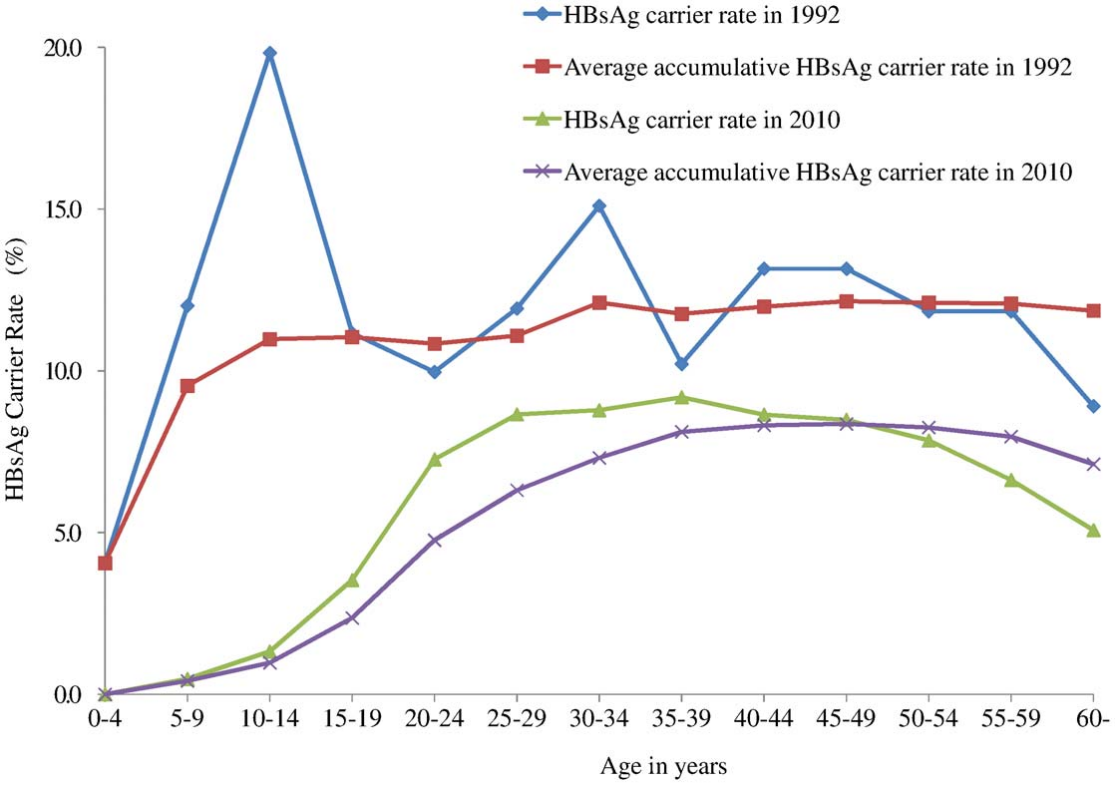
Comparison of HBsAg carrier rate and average cumulative HBsAg carrier rate by age (0 to 60 years) between 1992 and 2010. The blue line represents the carrier rate in different age groups in 1992, the red line the average cumulative carrier rate from zero to a specific age group in 1992, the green line the carrier rate in different age groups in 2010, and the purple line the average cumulative carrier rate from zero to a specific age group in 1992.

**Figure 3:**
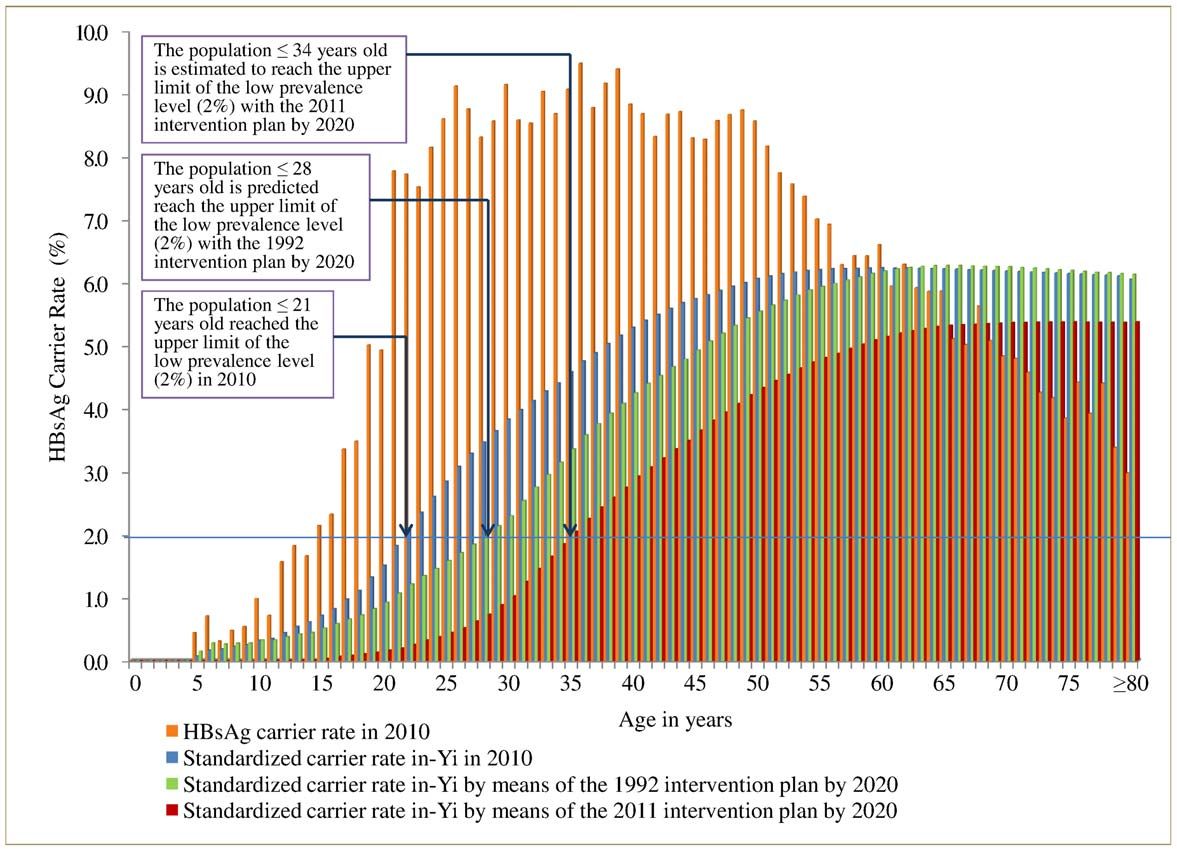
Comparison of predicted distribution of HBsAg carrier rate by age by the year 2020 under the 1992 and 2011Programs. The orange bar represents the real carrier rate by age, the blue bar the average cumulative carrier rate from zero to a specific age in 2010, the green bar the average cumulative carrier rate from zero to a specific age in 2020 under the 1992 Program, and the red bar the average cumulative carrier rate from zero to a specific age in 2020 with the 2011 Program. The line at 2% is the upper limit of the low prevalence level as defined by WHO.

Participants ≤20 years old had a significantly lower HBsAg carrier rate (1.51%) in 2010 than a similar group prior to 1992 (rate 9%–12%). From 1992 to 2010, the number of HBsAg carriers decreased by 1,577,391, a reduction of 47.20% for the standardized HBsAg carrier rate (11.61% in 1992 vs. 6.13% in 2010; Fig. 2). This implies that the vaccination program had saved US$17.8 billion in treating hepatitis B, the benefit: cost ratio of 124.9∶1 (Table 3 and Table S3). Further, we predict that the standardized HBsAg carrier rate in the whole provincial population will decrease from 6.13% in 2010 to 6.02% by 2020 under the 1992 Program and to 5.27% under the 2011Program. Using an HBsAg carrier rate of 2% as the upper limit of low prevalence according to the WHO criteria[18], with the 1992 Program, the population under 28 years would reach this goal, with a standardized HBsAg carrier rate of 1.97%. With the 2011 Program, the population under age 34 years would likewise be below the upper limit, with a standardized HBsAg carrier rate of 1.86% (Fig. 3). This implies that the 2011 Program will increase the age at which low prevalence is found from 28 to 34 years (Fig. 3 and Table S4).

**Table 3:** Cost and benefit analysis for 1992 and 2011 Programs.

	1992–2010	2010–2020	
Variables	Under 1992 Program	Under 1992 Program	Under 2011 Program
Total population across the province (×10^6^)	42.4	54.4	54.4
Reduced number of HBsAg carriers (×10^3^)	1,577.4	59.9	468.1
Reduced number of CHB(×10^3^)	157.7	6.0	46.8
Reduced number of patients with cirrhosis(×10^3^)	15.8	0.6	4.7
Reduced number of patients with HCC	1,577.4	59.9	468.1
Total utility in saving for CHB, cirrhosis and HCC (DALY, ×10^3^)	2,145.0	81.4	636.5
Total benefits in saving for CHB, cirrhosis and HCC ($US, ×10^6^)	17,763.5	674.2	5,271.1
Cost: effectiveness ratio*	50,075.7	588,590.0	292,666.1
Cost: utility ratio	36.8	432.8	215.2
Benefit: cost ratio	124.9	19.1	38.5

Notes: CHB  = chronic hepatitis B;HCC  =  hepatocellular carcinoma; DALY  = disability-adjusted life year; *: Cost for preventing one patient with HCC, 10 patients with cirrhosis, and 100 patients with CHB.

Moreover, compared with the 1992 Program, the 2011 Program is expected to significantly reduce the incidence of hepatitis B and the costs for its treatment. For example, under the 1992 Program, the number of HBsAg carriers would be reduced by 59,870, and with the 2011 Program, the number of HBsAg carriers would be reduced by 468,071 (Table S4). Thus, the HBsAg carrier reduction with the later program would be 7.82 times greater than that with the first program. We estimate it would cost US$137 million within the next 10 years to implement the 2011 Program in Zhejiang Province, but this expenditure would save US$5.27 billion for treating hepatitis B (Table 3 and Table S3), a benefit: cost ratio of 38.48∶1.

### Quality of Data from the EHR System

The data from the EHR system were accurate and reliable, as shown by comparison with the sampling survey (Fig. S3). The sex and age distributions in the two data resources were consistent.

## Discussion

Hepatitis B virus infection is endemic worldwide, but its prevalence differs greatly among regions[19], [20]. The last two surveys in China showed that the national prevalence of HBsAg was 8.75% in 1979[7] and 9.75% in 1992[8], making China a high endemic region. With the infant immunization beginning 1992, the prevalence of HBsAg was reduced to 7.18% in 2006[9]. However, this is still much higher than in Western countries, and a comprehensive prevention strategy is required[21]. An expanded population-based test for HBV infection is thus recommended [22].

By stratified analysis of community-based epidemiologic data, we identified several factors impacting the prevalence of HBV: male sex, being an adult aged ≥20 years, a family history of hepatitis B, drinking, smoking, being a migrant worker, and/or having some specific occupations (fishermen, construction worker, and long-distance truck driver). Chronic nicotine treatment increases the incidence of infection with several viruses, such as influenza[23], HIV-1[24], and HSV-1[25]. Although a high prevalence of HBsAg in long-distance truck drivers has never been reported before, a high incidence of HIV infection has been reported[26], which provides further support for our finding, as these viruses have similar infection modes[27].

Participants <20 years old had a significantly lower HBsAg carrier rate than other age groups. Most participants in this group were born after 1992, when the national HBV vaccination program for infants was implemented. National vaccination programs significantly decrease not only the HBV carrier rate but also HBV-associated complications[28], [29]. Although other factors, such as differences in population growth rate and increased investment and improved medical care structure, might affect the HBsAg rate, the reduction we observed provides supporting evidence for the effectiveness of the national vaccination program, which brought the HBsAg carrier rate to 1.51% by 2010. This is a highly significant reduction, as the rate was approximately 9%–12% for a similar age group prior to 1992[8] and equals a reduction of an estimated 1,577,391 HBsAg carriers and a savings of US$17.8 billion for treatment of HBV infections in Zhejiang Province. Likely reasons for the continued 1.51% HBsAg prevalence rate in young individuals are different vaccine coverage rates during early implementation and vaccine failure in some individuals.

To reduce the HBV infection rate in the older population, we implemented a comprehensive program for both infants and adults in 2011. Although immunization of targeted high-risk populations has been less successful in the US [2], it is too early to confirm the impact of the 2011 Program on HBV infection in China. However, considering the factors associated with the cost to a patient and different degrees of government involvement in the US and China, we expect the outcome in China would be very different. We predict that the 2011 Program will lead to a reduction of 468,071 HBV carriers and bring the HBsAg carrier rate among individuals under 34 years old to the low epidemic level of 2% or less by 2020. In contrast, with the 1992 Program, there would be a reduction of 59,870 HBV carriers and a change in the HBsAg carrier rate among individuals <28 years old to the low epidemic level by 2020. Thus, the 2011 Program would cause a 7.82-fold reduction in the number of carriers and expand the age range of those protected from 28 to 34 years. This is highly significant because this group is in their primary reproductive period[30], and a low HBsAg carrier rate (<2%), especially among prospective parents under 34 years old, implies fewer infants infected with HBV and parental transmission can be effectively controlled.

It appears that the new HBV vaccination program not only strengthened neonatal vaccination but also controlled HBV infection among women of childbearing age. Further, the 2011 Program would be expected to save US$5.27 billion for treating hepatitis B within the next 10 years, a benefit: cost ratio of 38.5∶1, under the same assumptions and approaches used in a previous study [17].

There are a few limitations of this study. First, anti-HBc and HBV DNA status were unavailable, there is significant fibrosis and inflammation in a small proportion of HBV-infected patients with persistently normal ALT, and an immune tolerance phase with a normal ALT and high HBV DNA often is found in the period of active reproduction in young women, which leads to much of the perinatal transmission[31]. Thus, the benefits of HBV vaccination could have been under estimated. Second, despite the safety and effectiveness of the hepatitis B vaccine and the benefits of government-oriented free programs, some potential barriers, included scheduling conflicts and forgetfulness, may influence the 2011 program, which may overestimate the effectiveness of the program[2], [32]. Third, we did not consider inflation and exchange rate differences between Chinese and US currency in our cost and benefit analyses, as we have no way to predict what could happen during this long period of time.

In conclusion, by analyzing large-scale epidemiologic data on hepatitis B in Zhejiang Province of China, we identified several factors impacting HBV infection. The 1992 Program has contributed greatly to a reduced HBsAg carrier rate in the young. Further, our modelling suggested that the 2011 Program not only reduced the costs associated with hepatitis B treatment, but also brought the HBsAg carrier rate among people under the age of 34 years to 2%, expanding the age of protection from 28 years under the 1992 Program to 34 years under the 2011 Program. This is highly significant, as this age group is highly active reproductively, and a reduced HBV infection rate would mean a reduction of parental transmission. It is our hope that the findings from this study can be used to support application of the new HBV vaccination program to other high and middle endemic regions of China and other countries fighting hepatitis B.

## Supporting Information

Figure S1 **Distribution of prevalence of suspected hepatitis B in different age groups.** The data were from a community-based epidemiologic survey (N = 761,544). The prevalence of suspected hepatitis B was highest among participants aged 20–49 years and showed a trend to a sharp decrease from 20 years to zero years old.
(TIF)

Figure S2 **Relation of HBsAg carrier rate and the prevalence of suspected hepatitis B in the coastal regions and fisherman.** (A) Carrier rate by age, sex, and district (coastal regions vs. inland). (B) Prevalence of suspected hepatitis B by age, sex, and district (coastal regions vs. inland). (C) Carrier rate by age, sex, and occupation (fishermen vs. non-fishermen). (D) Prevalence of suspected hepatitis B by age, sex, and occupation (fishermen vs. non-fishermen).
(TIF)

Figure S3 **Comparison of sex (A) and age (B) distribution of HBsAg carrier rate in the community-based EHR system and epidemiologic sampling survey.**
(TIF)

Table S1 **The formula for the reduced number of CHB, cirrhosis and HCC, the cost of the treatment, and the money saved.**
(DOC)

Table S2 **Influencing factors on the prevalence of suspected hepatitis B for ages from 20–60 years.**
(DOC)

Table S3 **Cost and benefit analysis for the 1992 and 2011 intervention plan for hepatitis B.**
(DOC)

Table S4 **Prediction of HBsAg carrier rate in the year 2020.**
(DOC)
