# Supplementary figures and images for: Effectiveness of HBV Vaccination in Infants and Prediction of HBV Prevalence Trend under New Vaccination Plan: Findings of a Large-Scale Investigation
Source: PLoS One. 2012 Oct 19;7(10):e47808. doi: 10.1371/journal.pone.0047808 (PMC3477110; doi:10.1371/journal.pone.0047808)

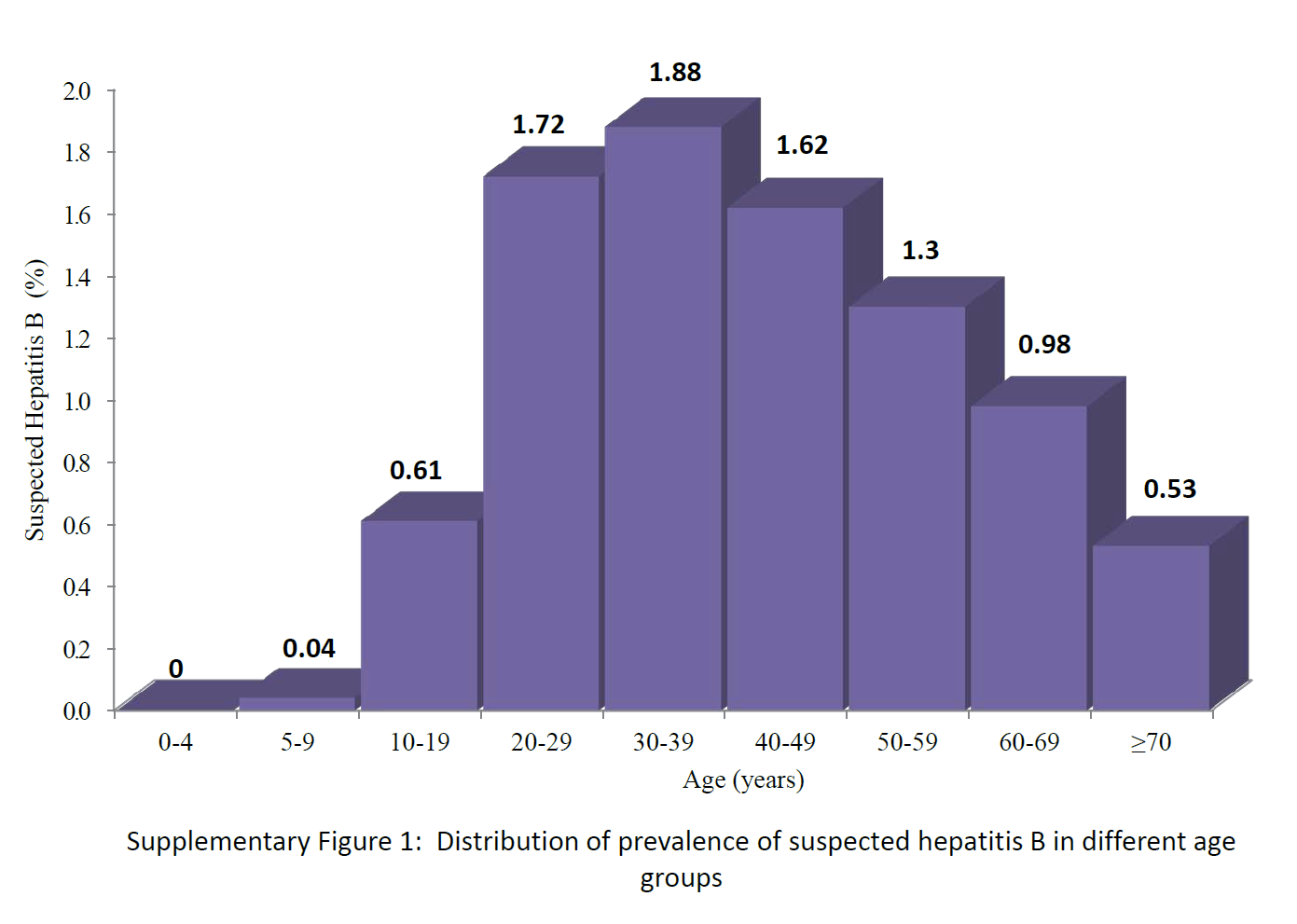

Supplement: Figure S1 — Distribution of prevalence of suspected hepatitis B in different age groups. The data were from a community-based epidemiologic survey (N = 761,544). The prevalence of suspected hepatitis B was highest among participants aged 20–49 years and showed a trend to a sharp decrease from 20 years to zero years old. (TIF) [file pone.0047808.s001.tif]

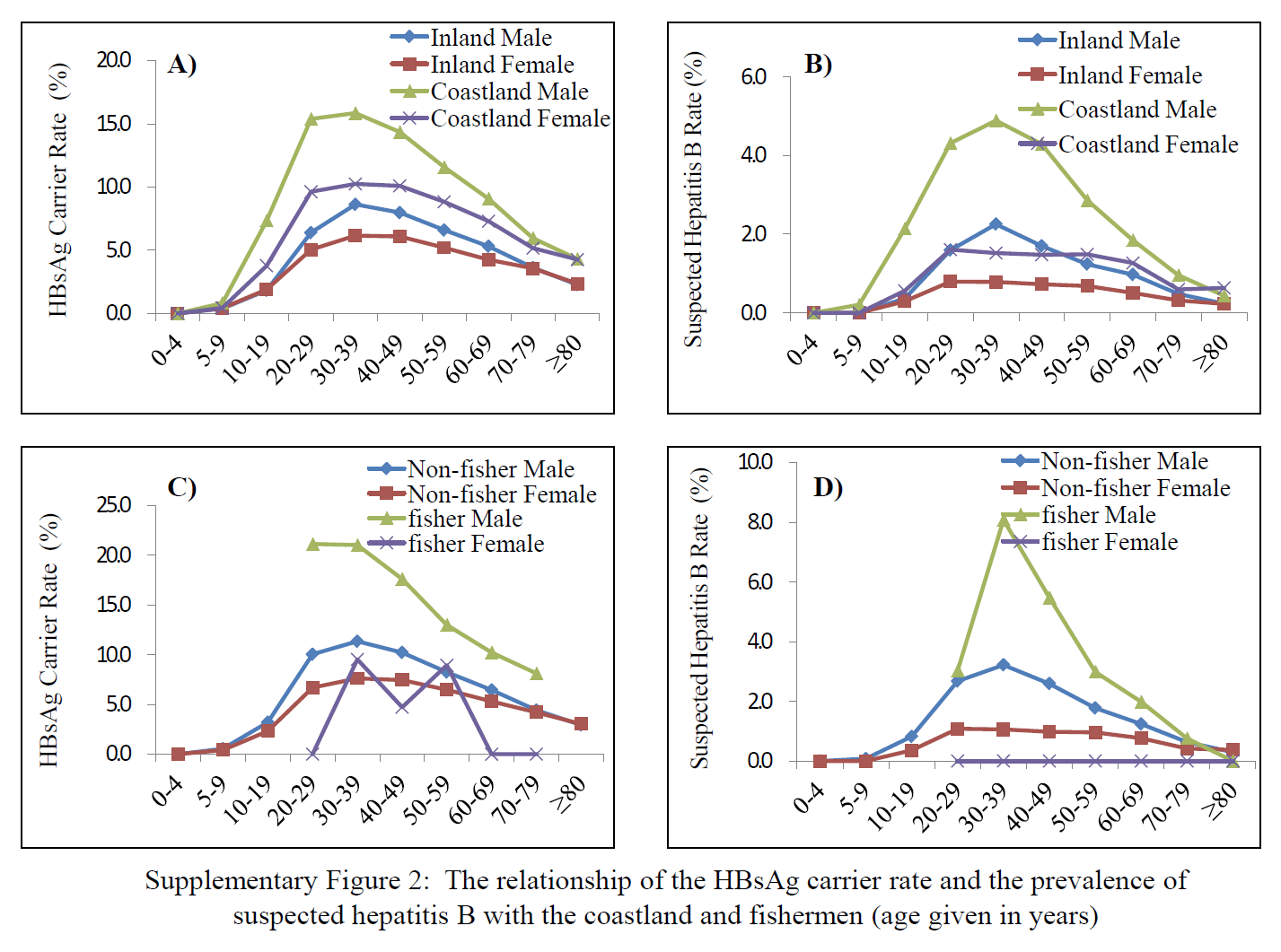

Supplement: Figure S2 — Relation of HBsAg carrier rate and the prevalence of suspected hepatitis B in the coastal regions and fisherman. (A) Carrier rate by age, sex, and district (coastal regions vs. inland). (B) Prevalence of suspected hepatitis B by age, sex, and district (coastal regions vs. inland). (C) Carrier rate by age, sex, and occupation (fishermen vs. non-fishermen). (D) Prevalence of suspected hepatitis B by age, sex, and occupation (fishermen vs. non-fishermen). (TIF) [file pone.0047808.s002.tif]

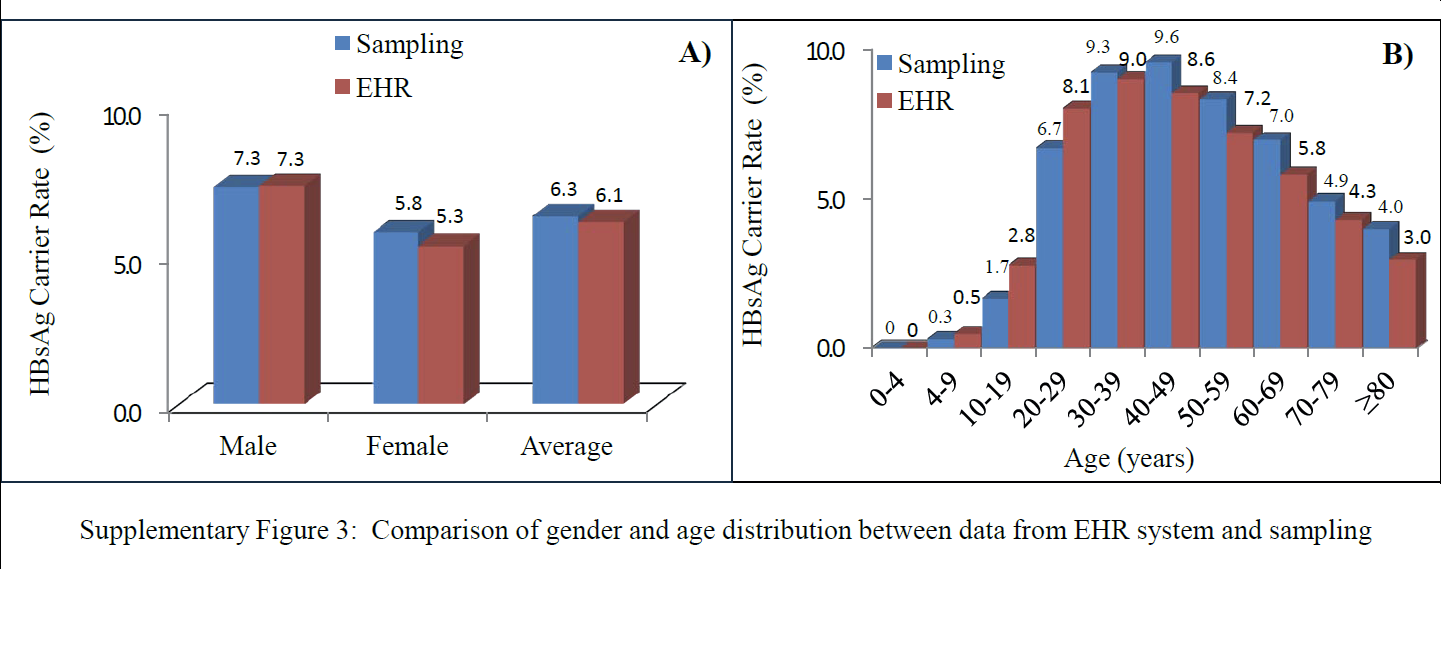

Supplement: Figure S3 — Comparison of sex (A) and age (B) distribution of HBsAg carrier rate in the community-based EHR system and epidemiologic sampling survey. (TIF) [file pone.0047808.s003.tif]
