# Supplementary material for: Effectiveness of HBV Vaccination in Infants and Prediction of HBV Prevalence Trend under New Vaccination Plan: Findings of a Large-Scale Investigation
Source: PLoS One. 2012 Oct 19;7(10):e47808. doi: 10.1371/journal.pone.0047808 (PMC3477110; doi:10.1371/journal.pone.0047808)
Supplement: Table S1 — The formula for the reduced number of CHB, cirrhosis and HCC, the cost of the treatment, and the money saved. (DOC) [file pone.0047808.s004.doc]

Supplementary Table 1. the formula for the reduced number of CHB, cirrhosis and HCC, the cost of the treatment, and the money saved

|  | **1992–2010** | **2010–2020** | |
| --- | --- | --- | --- |
| **Variables** | Under 1992Program | Under 1992Program | Under 2011 Program |
| **Total population across the province** | Total population across the province in1992 | Total population across the province in 2010 | Total population across the province in 2010 |
| **Reduced number of HBsAg carriers** | The number of HBsAg carriers in 1992 - The number of HBsAg carriers in 2010 under 1992 Program | The number of HBsAg carriers in 2010 - The number of HBsAg carriers in 2020 under 1992 Program | The number of HBsAg carriers in 2010 - The number of HBsAg carriers in 2020 under 2011 Program |
| **Reduced number of CHB** | Reduced number of HBsAg carriers from 1992 to 2010 under 1992 Program × 0.1 | Reduced number of HBsAg carriers from 2011 to 2020 under 1992 Program × 0.1 | Reduced number of HBsAg carriers from 2011 to 2020 under 2011 Program × 0.1 |
| **Reduced number of patients with cirrhosis** | Reduced number of CHB from 1992 to 2010 under 1992 Program × 0.1 | Reduced number of CHB from 2011 to 2020 under 1992 Program × 0.1 | Reduced number of CHB from 2011 to 2020 under 2011 Program × 0.1 |
| **Reduced number of patients with HCC** | Reduced number of patients with cirrhosis from 1992 to 2010 under 1992 Program × 0.1 | Reduced number of patients with cirrhosis from 2011 to 2020 under 1992 Program × 0.1 | Reduced number of patients with cirrhosis from 2011 to 2020 under 2011 Program × 0.1 |
| **Total utility in saving for CHB, cirrhosis and HCC (DALY,×103)** | Reduced number of CHB from 1992 to 2010 × 11.68+ Reduced number of patients with cirrhosis from 1992 to 2010 × 17.57+ Reduced number of patients with HCC from 1992 to 2010 × 16.11 | Reduced number of CHB from 2011 to 2020 under 1992 Program × 11.68+ Reduced number of patients with cirrhosis from 2011 to 2020 under 1992 Program × 17.57+ Reduced number of patients with HCC from 2011 to 2020 under 1992 Program × 16.11 | Reduced number of CHB from 2011 to 2020 under 2011 Program × 11.68+ Reduced number of patients with cirrhosis from 2011 to 2020 under 2011 Program × 17.57+ Reduced number of patients with HCC from 2011 to 2020 under 2011 Program × 16.11 |
| **Total benefits in saving for CHB, cirrhosis and HCC** | Reduced number of CHB from 1992 to 2010 × 105297.8 USD + Reduced number of patients with cirrhosis from 1992 to 2010 ×65830.7 USD + Reduced number of patients with HCC from 1992 to 2010 ×73228.8 USD | Reduced number of CHB from 2011 to 2020 under 1992 Program × 105297.8 USD + Reduced number of patients with cirrhosis from 2011 to 2020 under 1992 Program × 65830.7 USD + Reduced number of patients with HCC from 2011 to 2020 under 1992 Program × 73228.8 USD | Reduced number of CHB from 2011 to 2020 under 2011 Program × 105297.8 USD + Reduced number of patients with cirrhosis from 2011 to 2020 under 2011 Program × 65830.7 USD + Reduced number of patients with HCC from 2011 to 2020 under 2011 Program ×73228.8 USD |
| **Cost: effectiveness ratio** | Cost: effectiveness ratio = total cost for testing and vaccination/reduced number of patients with chronic hepatitis B (CHB), cirrhosis, or hepatocellular carcinoma (HCC); | | |
| **Cost: utility ratio** | cost: utility ratio = total cost for testing and vaccination/avoided disability-adjusted life years (DALY) | | |
| **Benefit: cost ratio** | benefit: cost ratio = total benefits in saving treatment for CHB, cirrhosis, and HCC/total cost for testing and vaccination. | | |
