# Supplementary material for: Effectiveness of HBV Vaccination in Infants and Prediction of HBV Prevalence Trend under New Vaccination Plan: Findings of a Large-Scale Investigation
Source: PLoS One. 2012 Oct 19;7(10):e47808. doi: 10.1371/journal.pone.0047808 (PMC3477110; doi:10.1371/journal.pone.0047808)
Supplement: Table S2 — Influencing factors on the prevalence of suspected hepatitis B for ages from 20–60 years. (DOC) [file pone.0047808.s005.doc]

Supplementary Table 2: Influencing factors on the prevalence of suspected hepatitis B for ages from 20-60 years

| Factors | Standardized rate (%) of suspected HB (N) | | RR | 95% CI | ARP* (%) | P value |
| --- | --- | --- | --- | --- | --- | --- |
|  | Exposure | Non-exposure |  |  |  |  |
| Long-distance truck driver | 1.95 (697) | 1.72 (607,617) | 1.17 | 0.7-1.96 | 13.37 | 0.5581 |
| Construction worker | 2.84 (902) | 1.72 (607,412) | 1.68 | 1.15-2.45 | 65.12 | 0.0073 |
| Floating population | 1.37 (2,741) | 1.35 (556,107) | 1.03 | 0.75-1.41 | 1.48 | 0.8670 |
| With family history | 2.4 (13,110) | 1.38 (408,887) | 1.74 | 1.56-1.95 | 73.91 | <0.0001 |
| Smoking | 2.91 (67,091) | 1.59 (277,937) | 1.83 | 1.74-1.93 | 83.02 | <0.0001 |
| Drinking | 3.04 (58,830) | 1.61 (285,545) | 1.89 | 1.79-1.99 | 88.82 | <0.0001 |
| Residents in coastal regions | 2.07 (237,139) | 0.87 (467,536) | 2.38 | 2.28-2.48 | 137.93 | <0.0001 |

* Notes: ARP (Attributable risk proportion) = proportion of increased risk attributing to the given factor.
