# Supplementary material for: Effectiveness of HBV Vaccination in Infants and Prediction of HBV Prevalence Trend under New Vaccination Plan: Findings of a Large-Scale Investigation
Source: PLoS One. 2012 Oct 19;7(10):e47808. doi: 10.1371/journal.pone.0047808 (PMC3477110; doi:10.1371/journal.pone.0047808)
Supplement: Table S3 — Cost and benefit analysis for the 1992 and 2011 intervention plan for hepatitis B. (DOC) [file pone.0047808.s006.doc]

Supplementary Table 3: Cost and benefit analysis for the 1992 and 2011 intervention plan for hepatitis B

|  | 1992 to 2010 |  | 2010 to 2020 | |
| --- | --- | --- | --- | --- |
| Variables | Under the 1992 Intervention plan |  | Under the 1992 Intervention plan | Under the 2011 Intervention plan |
| Total population (x10^6^) | 42.4 |  | 54.4 | 54.4 |
| Number of HBsAg carriers (x10^3^) | 4,913.8 |  | 3,336.4 | 3,336.4 |
| **Cost (US $)** |  |  |  |  |
| Total cost for HBsAg tests (x10^6^) | 0.0 |  | 0.0 | 14.8 |
| Total cost for HBsAb tests (x10^6^) | 0.0 |  | 0.0 | 15.0 |
| Number of people vaccinated with HBV vaccine (x10^6^) | 15.0 |  | 6.7 | 19.4 |
| Total cost for HBV vaccine (x10^6^) | 79.0 |  | 35.2 | 107.2 |
| Total cost for testing and vaccination (x10^6^) | 79.0 |  | 35.2 | 137.0 |
| **Cost effectiveness analysis** |  |  |  |  |
| Reduced number of HBsAg carriers (x10^3^) | 1,577.4 |  | 59.9 | 468.1 |
| Reduced umber of CHB (x10^3^) | 157.7 |  | 6.0 | 46.8 |
| Reduced number of patients with cirrhosis (x10^3^) | 15.8 |  | 0.6 | 4.7 |
| Reduced number of patients with HCC | 1,577.4 |  | 59.9 | 468.1 |
| Cost effectiveness ratio* | 50,075.7 |  | 588,590.0 | 292,666.1 |
| **Cost utility analysis (DALY)** |  |  |  |  |
| The utility in treatment saving for 1 CHB patient | 11.7 |  | 11.7 | 11.7 |
| The utility in treatment saving for 1 cirrhosis | 17.6 |  | 17.6 | 17.6 |
| The utility in treatment saving for 1 HCC | 16.1 |  | 16.1 | 16.1 |
| Total utility in treatment saving for 1 CHB patient (x10^3^) | 1,842.4 |  | 69.9 | 546.7 |
| Total utility in treatment saving for 1 cirrhosis (x10^3^) | 277.1 |  | 10.5 | 82.2 |
| Total utility in treatment saving for 1 HCC (x10^3^) | 25.4 |  | 1.0 | 7.5 |
| Total utility in treatment saving for CHB, cirrhosis & HCC (x10^3^) | 2,145.0 |  | 81.4 | 636.5 |
| Cost utility ratio | 36.8 |  | 432.8 | 215.2 |
| **Cost benefit analysis (US $)** |  |  |  |  |
| The benefits in treatment saving for 1 CHB patient (x10^3^) | 105.3 |  | 105.3 | 105.3 |
| The benefits in treatment saving for 1 cirrhosis (x10^3^) | 65.8 |  | 65.8 | 65.8 |
| The benefits in treatment saving for 1 HCC (x10^3^) | 73.2 |  | 73.2 | 73.2 |
| Total benefits in treatment saving for CHB patients (x10^6^) | 16,609.6 |  | 630.4 | 4,928.7 |
| Total benefits in treatment saving for cirrhosis (x10^6^) | 1,038.4 |  | 39.4 | 308.1 |
| Total benefits in treatment saving for HCCs (x10^6^) | 115.5 |  | 4.4 | 34.3 |
| Total benefits in treatment saving for CHB, cirrhosis & HCC (x10^6^) | 17,763.5 |  | 674.2 | 5,271.1 |
| Benefit-cost ratio | 124.9 |  | 19.1 | 38.5 |

Notes: CHB = chronic hepatitis B patients; HCC = hepatocellular carcinoma; DALY = Disability-adjusted life year; *: Cost for reducing one patient with HCC, 10 patients with cirrhosis, 100 patients with CHB.
