# Supplementary material for: Effectiveness of HBV Vaccination in Infants and Prediction of HBV Prevalence Trend under New Vaccination Plan: Findings of a Large-Scale Investigation
Source: PLoS One. 2012 Oct 19;7(10):e47808. doi: 10.1371/journal.pone.0047808 (PMC3477110; doi:10.1371/journal.pone.0047808)
Supplement: Table S4 — Prediction of HBsAg carrier rate in the year 2020. (DOC) [file pone.0047808.s007.doc]

Supplementary Table 4: Prediction of HBsAg carrier rate in the year 2020

| Age  (*Yi*) | HBsAg carrier rate (%) | Crude carrier rate *in-Yi* (%) | Standardized carrier rate *in-Yi* in 2010 (%) | Number of carriers *in-Yi* in 2010 | Standardized carrier rate *in-Yi* with the 1992 intervention plan by 2020 (%) | Number of carriers *in-Yi* with the 1992 intervention plan by 2020 | Standardized carrier rate *in-Yi* with the 2011 intervention plan by 2020 (%) | Number of carrier rate *in-Yi* with the 2011 intervention plan by 2020 (%) |
| --- | --- | --- | --- | --- | --- | --- | --- | --- |
| 5 | 0.45 | 0.17 | 0.08 | 2,798 | 0.15 | 5,596 | 0.00 | 0 |
| 10 | 0.99 | 0.56 | 0.32 | 22,228 | 0.33 | 22,641 | 0.01 | 413 |
| 15 | 2.14 | 1.15 | 0.72 | 73,278 | 0.51 | 52,174 | 0.03 | 3,322 |
| 20 | 4.92 | 2.74 | 1.51 | 205,935 | 0.93 | 126,232 | 0.17 | 23,264 |
| 25 | 8.60 | 5.13 | 2.84 | 487,958 | 1.59 | 272,624 | 0.45 | 77,441 |
| 30 | 9.15 | 6.60 | 3.83 | 787,181 | 2.30 | 473,466 | 1.03 | 211,072 |
| 35 | 9.07 | 7.49 | 4.58 | 1,108,805 | 3.36 | 814,170 | 2.05 | 497,368 |
| 40 | 8.83 | 8.19 | 5.29 | 1,517,585 | 4.25 | 1,219,407 | 2.93 | 840,011 |
| 45 | 8.30 | 8.31 | 5.74 | 1,917,100 | 4.92 | 1,644,152 | 3.65 | 1,218,130 |
| 50 | 8.56 | 8.37 | 6.06 | 2,276,207 | 5.54 | 2,083,304 | 4.33 | 1,628,063 |
| 55 | 7.01 | 8.20 | 6.20 | 2,582,380 | 5.93 | 2,469,643 | 4.81 | 2,000,120 |
| 60 | 6.60 | 7.92 | 6.23 | 2,812,409 | 6.18 | 2,791,480 | 5.15 | 2,322,745 |
| 65 | 5.86 | 7.70 | 6.22 | 2,952,027 | 6.27 | 2,977,857 | 5.32 | 2,523,699 |
| 70 | 4.83 | 7.52 | 6.19 | 3,027,284 | 6.25 | 3,057,153 | 5.36 | 2,624,683 |
| 75 | 3.84 | 7.33 | 6.14 | 3,082,710 | 6.19 | 3,109,420 | 5.37 | 2,698,391 |
| All age groups | 1.99 | 7.12 | 6.13 | 3,336,369 | 6.02 | 3,276,499 | 5.27 | 2,868,298 |
